# Reduction in Measurement Error Confounds Cumulative Pollution Exposure

**DOI:** 10.1289/ehp.11804

**Published:** 2008-10

**Authors:** Adrian Barnett

**Affiliations:** School of Public Health, Queensland University of Technology, Kelvin Grove, Queensland, Australia, E-mail: a.barnett@qut.edu.au

Barraza-Villarreal et al. (2008) showed a convincing link between increased air pollution and reduced forced expiratory volume in 1 sec (FEV_1_). However, the apparent stronger association between reduced FEV_1_ and cumulative exposure over 1–5 days may be due in part to a reduction in measurement error of particulate matter < 2.5 μm (PM_2.5_) and not a true cumulative effect (Barraza-Villarreal et al.’s Figure 3).

Air pollution studies are prone to measurement error. In the study of Barraza-Villarreal et al. (2008)—as in most others—the estimates of air pollution came from a network of fixed monitors. Each child’s day-to-day exposure was assigned using the closest monitor, and no monitors were > 5 km from the child’s home or school. However, even with a monitor near the child’s location, the estimate cannot be perfect because of variation in individual exposure (e.g., time spent outdoors).

I evaluated the effect of measurement error using a simulation study. I assumed that the 158 asthmatic children had a PM_2.5_ exposure given by


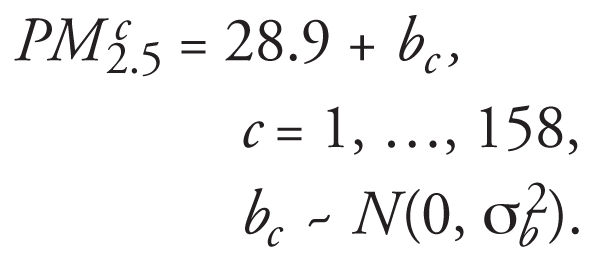


The mean PM_2.5_ exposure is 28.9 μg/m3, and each child (*c*) varies around this mean (*b*). This between-child variation means that some children live in more polluted areas than others.

The children’s FEV_1_ was observed at repeated times, which was simulated using


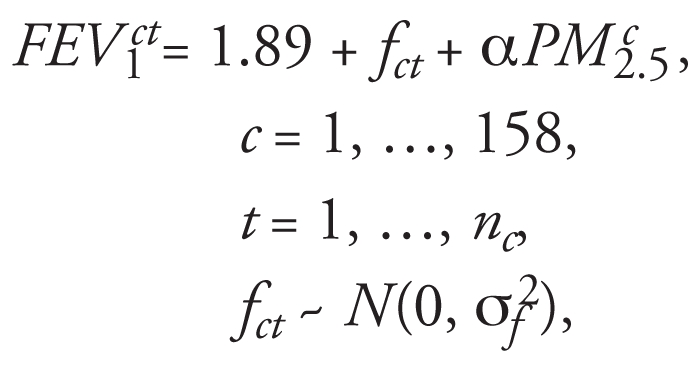


where 1.89 L/sec is the mean FEV_1_, *t* is time, *n_c_* is the number of observations for child *c*, and *f_ct_* is the measurement error in FEV_1_. The parameter α controls the change in FEV_1_ due to PM_2.5_ exposure.

In the study of Barraza-Villarreal et al. (2008), FEV_1_ was dependent on PM_2.5_ exposure from the previous 1–5 days. Daily PM_2.5_ values are subject to measurement error (*e*), which I simulated using


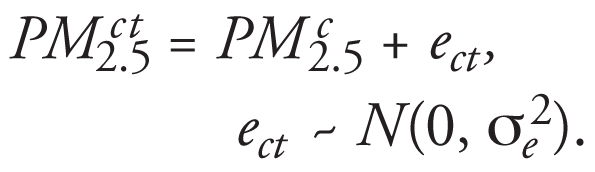


Barraza-Villarreal et al. (2008) used a mixed model to estimate the effect of PM_2.5_ on FEV_1_ and controlled for the repeated FEV_1_ results from the same child. They also controlled for a number of covariates; however, for this simulation study I simply regressed the simulated daily values, *FEV*_1_*^ct^*, against the simulated daily pollution values, *PM*_2.5_*^ct^* , and included a random intercept for each child.

I assumed a between-child variation in PM_2.5_ of σ*_b_*^2^ = 2.82 and an equal measurement error in PM_2.5_ of σ*_e_*^2^ = 2.82 (by naively using the standard deviation in PM_2.5_). I assumed a measurement error variation in FEV_1_ of σ*_e_*^2^ = 0.662. I simulated data for 158 children and random sampled the number of observations per child (*n_c_*) by rounding a randomly generated value from a normal distribution *N*(11,2.2^2^).

The results of 100 simulations are shown in Figure 1. Longer exposure lags gave estimated reductions that more closely approximated the true effect. On face value, longer exposure appears to be more damaging to health, but the simulated data had no cumulative effect. The stronger effect occurred because of the regression dilution bias and a reduction in the measurement error of PM_2.5_ exposure from using multiple days (MacMahon et al. 1990). Although different simulation results can be obtained by varying the strength of the pollution effect and measurement errors, the trend will always be to increased effects with increasing exposure periods.

The results of this simulation show that care should be taken when summing repeated measurements. Cumulative measurements are confounded by reductions in measurement error, which makes interpretation difficult.

The results of this simulation in no way invalidate the results found by Barraza-Villarreal et al. (2008). There is strong evidence that increased exposure to air pollution damages lung function. However, it is difficult to estimate how much of this reduction is due to a cumulative effect, thus requiring methodological development.

## Figures and Tables

**Figure 1:**
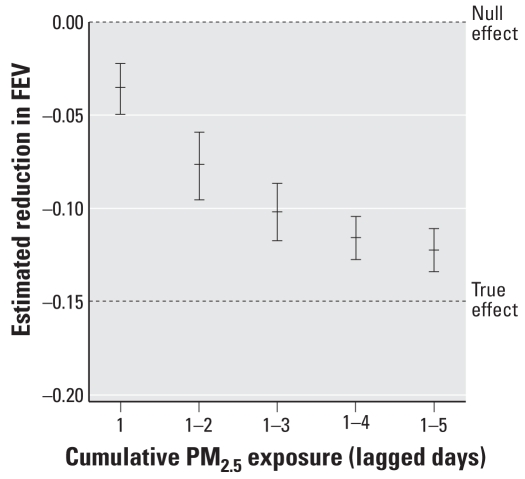
Increase in the estimated effect of PM_2.5_ with increasing lag using a simulation study. Vertical lines are the mean estimate and 95% confidence interval.
